# Supplementary material for: TEAD-independent mechanisms of YAP function in cardiomyocyte cell cycle reentry
Source: Life Sci Alliance. 2026 Jan 23;9(4):e202503496. doi: 10.26508/lsa.202503496 (PMC12830084; doi:10.26508/lsa.202503496)

**Figure 1:**


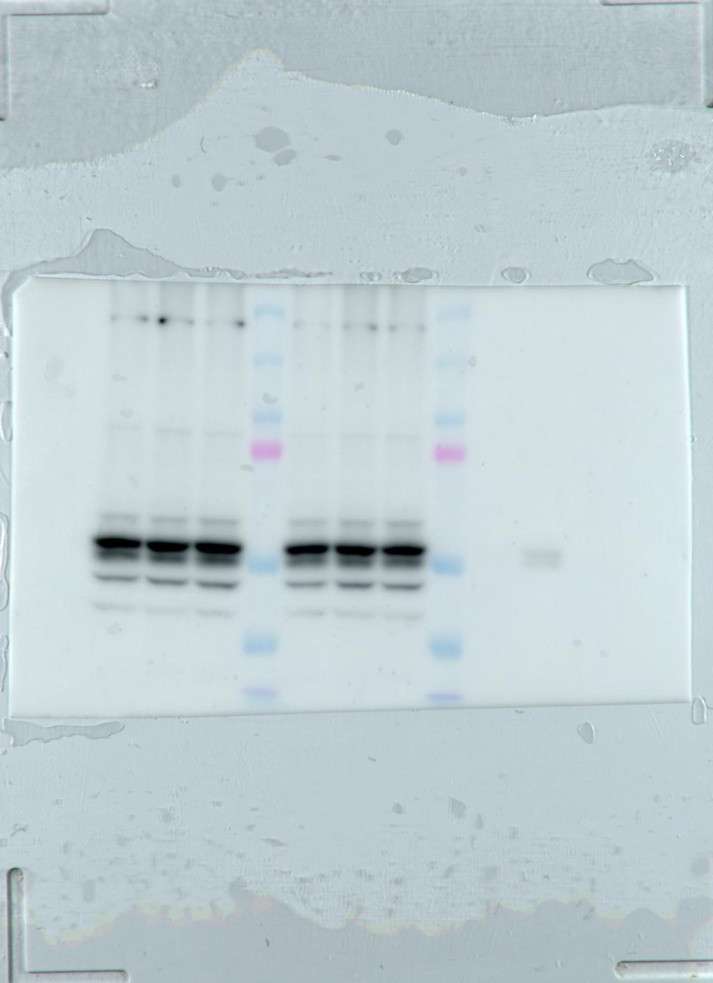

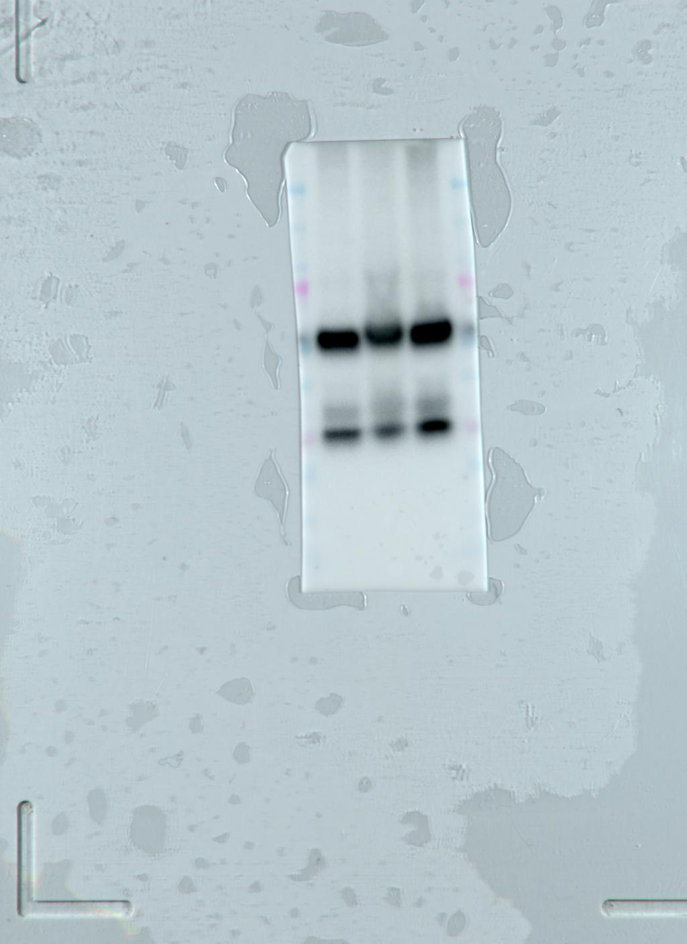

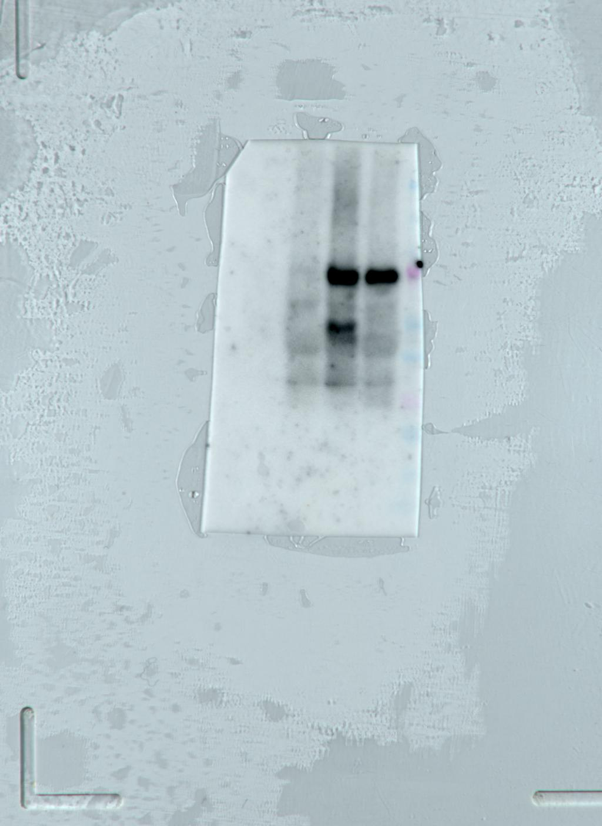
Flag: Actin: TEADs: (input + co-IP)

co-IP output:


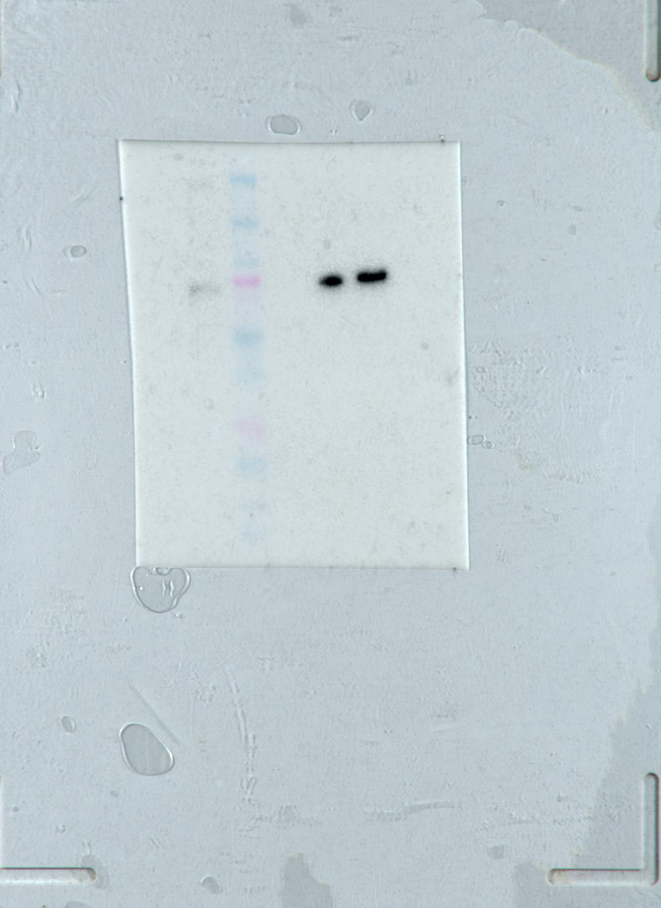


**Figure 5:**

Figure 5C:


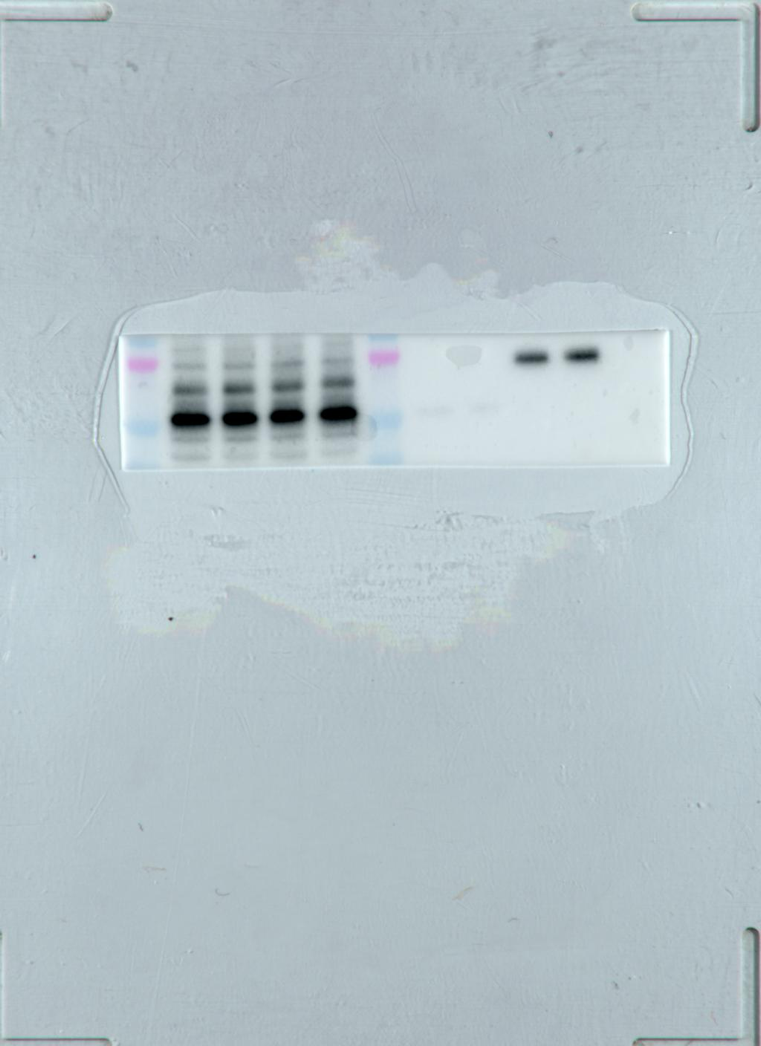
MPDZ: TEADs:


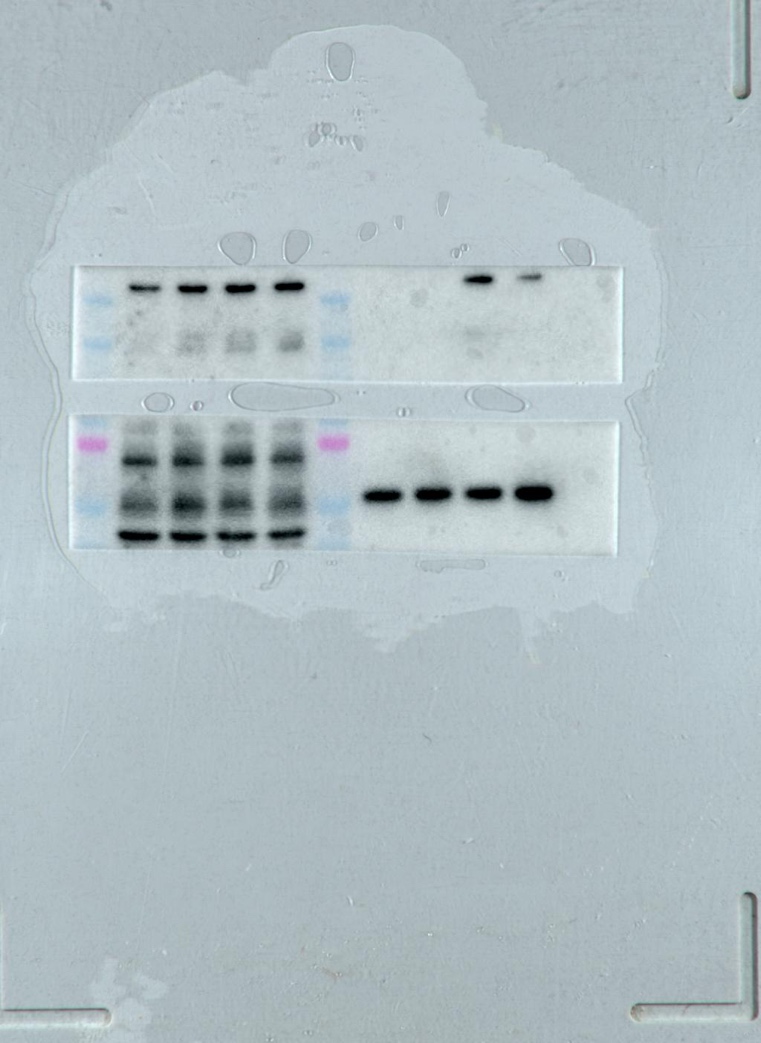


YAP:


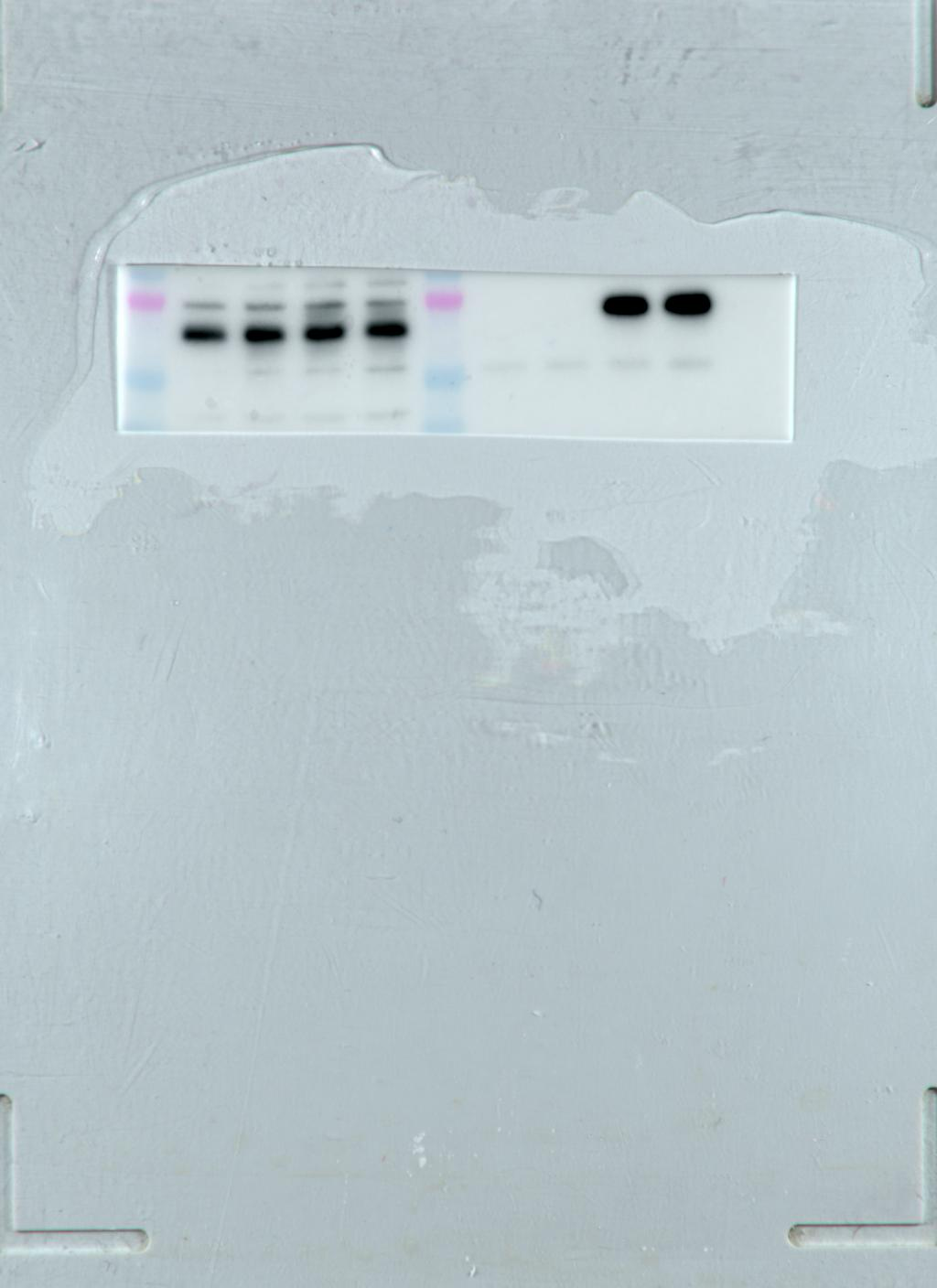


Figure 5F:

GAPDH: MPDZ: RhoA/B/C:


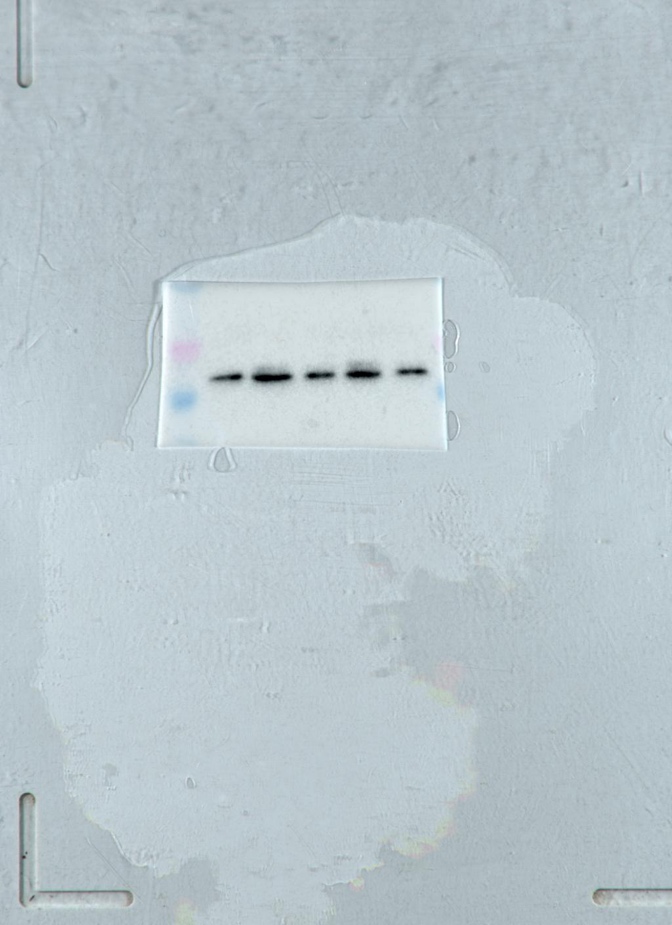

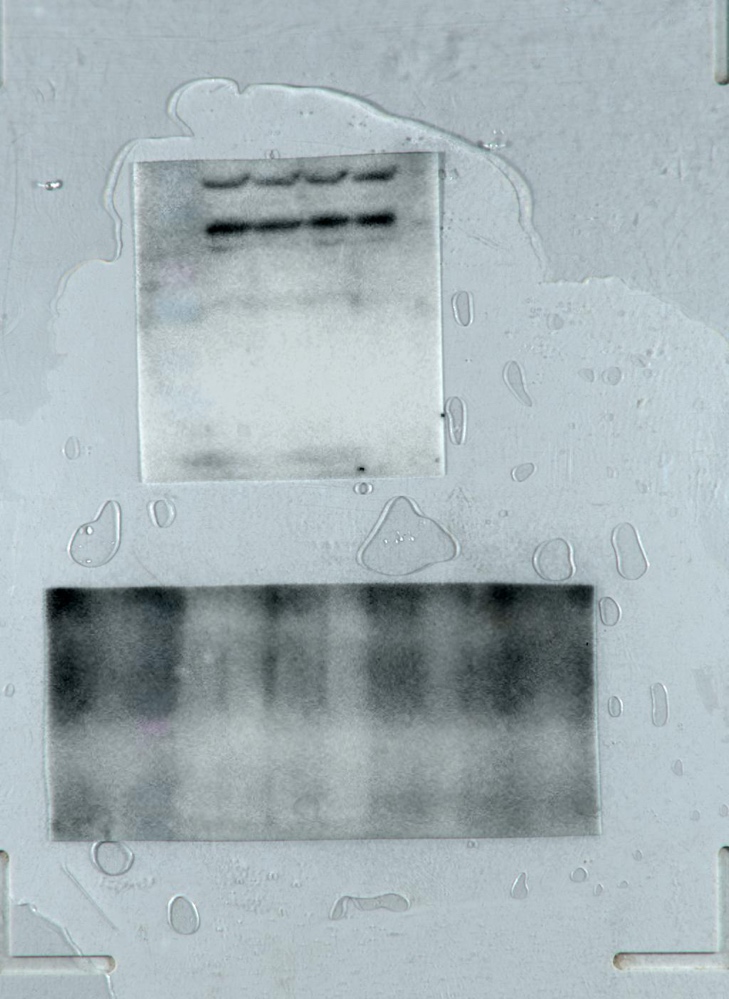

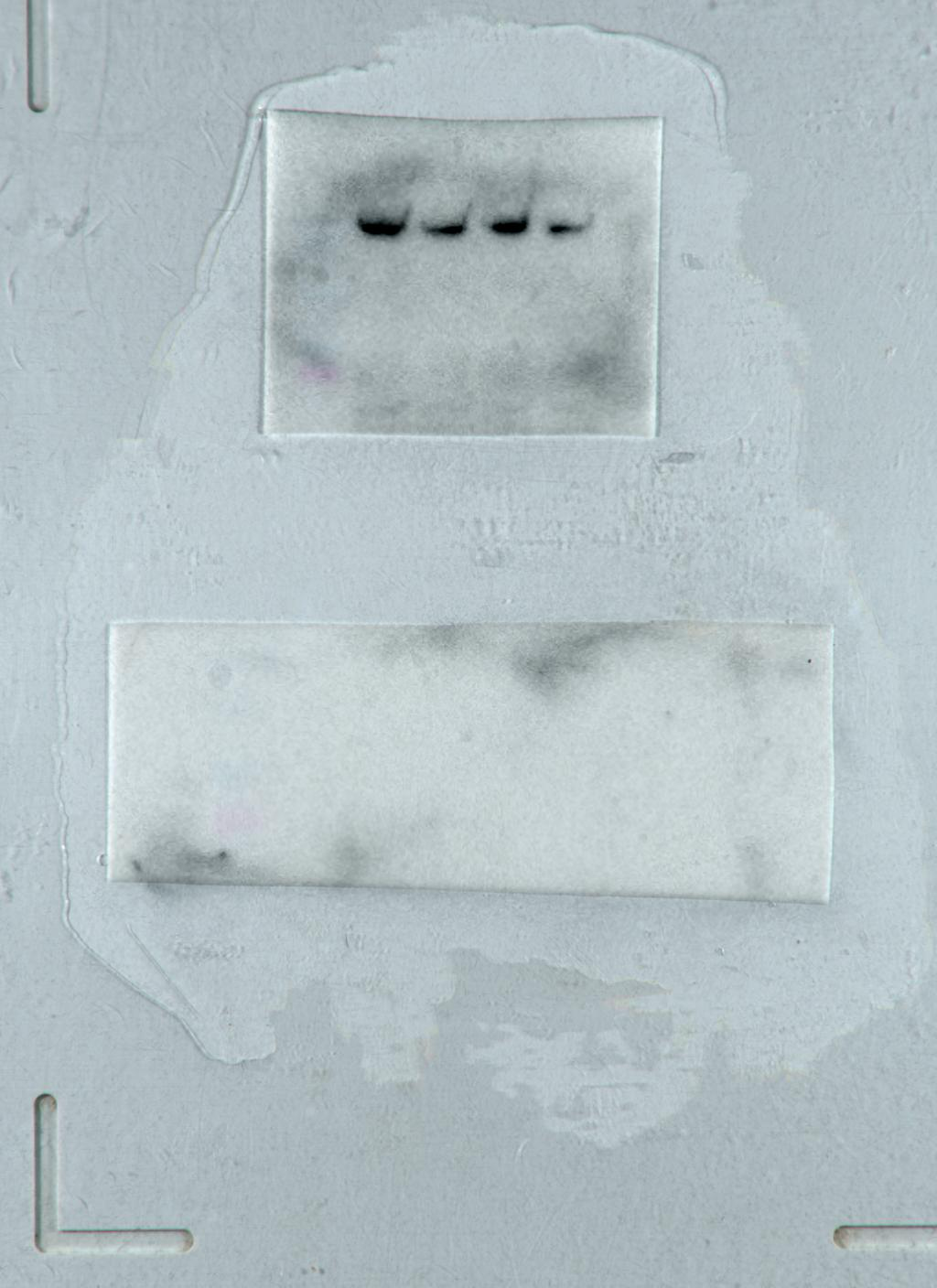

Supplement: Supplementary file 1 [file LSA-2025-03496_SdataF1_F5.1.docx]
